# Parameter zur Messung der regionalen Versorgungssituation: Ein Vergleich verschiedener Versorgungsparameter am Beispiel der augenärztlichen Versorgung in der Metropolregion Hamburg

**DOI:** 10.1007/s00347-020-01242-y

**Published:** 2020-10-27

**Authors:** A. Kis, J. Augustin, T. Lischka

**Affiliations:** 1 Institut für Versorgungsforschung in der Dermatologie und bei Pflegeberufen (IVDP), Competenzzentrum Versorgungsforschung in der Dermatologie (CVderm), Universitätsklinikum Hamburg-Eppendorf (UKE), Martinistr. 52, 20246 Hamburg, Deutschland; 2 Universitätsklinik für Augenheilkunde, Pius-Hospital Oldenburg, Georgstr. 12, 26121 Oldenburg (Oldb), Deutschland

**Keywords:** Erreichbarkeit, Versorgungsgrad, Orthoptist, Bedarfsplanung, Gesundheitsversorgung, Accessibility, Adjusted supply rate, Orthoptist, Requirement planning, Healthcare supply

## Abstract

**Hintergrund:**

Die regionale augenärztliche Versorgung wird in Deutschland über die Bedarfsplanungs-Richtlinie mit dem korrigierten Versorgungsgrad gesteuert. Der korrigierte Versorgungsgrad ist ein Instrument, um die medizinische Versorgungssituation abzubilden, allerdings haben frühere Studien Grenzen offenbart. So werden Faktoren wie Erreichbarkeit des öffentlichen Personennahverkehrs (ÖPNV) oder das Vorhandensein von Gesundheitsfachberufen zur Bewertung der Versorgungssituation noch nicht im ausreichenden Maße berücksichtigt. Insbesondere Gesundheitsfachberufe haben eine starke unterstützende Funktion in der regionalen Gesundheitsversorgung.

**Ziel der Arbeit:**

Ziel dieser Studie ist es, verschiedene Parameter zur Überprüfung der regionalen Gesundheitsversorgung am Beispiel der ophthalmologischen Versorgung kritisch zu vergleichen.

**Material und Methoden:**

Hierfür wurde exemplarisch für die Metropolregion Hamburg ein Score aus den Parametern „Dichte der Arztstandorte mit orthoptischem Angebot (Angebote/100.000 Einwohner) auf Kreisebene“, der „Bevölkerungsanteil mit ÖPNV-Erreichbarkeit zum nächstgelegenen Augenarzt <30 min auf Kreisebene“, der „Bevölkerungsanteil mit ÖPNV-Erreichbarkeit zum nächstgelegenen Augenarzt mit orthoptischem Angebot <30 min auf Kreisebene“ gebildet und dem korrigierten Versorgungsgrad gegenübergestellt.

**Ergebnisse:**

Während der korrigierte Versorgungsgrad in keinem der Teilräume eine Unterversorgung feststellen kann, offenbaren die anderen betrachteten Parameter durchaus deutliche Versorgungsunterschiede.

**Diskussion:**

Die Betrachtung unterschiedlicher Parameter zeigt ein heterogenes Bild der Versorgungssituation. Dies sollte in der Bedarfsplanung für die Bewertung berücksichtigt werden.

## Abstract (en)

**Background:**

Regional ophthalmologic care in Germany is ensured through the guidelines of the Federal Joint Committee’s requirement planning and the adjusted supply rate defined therein. Although the adjusted supply rate is an official instrument for assessing the actual healthcare situation in Germany, earlier studies revealed limitations. Factors such as public transport accessibility or the existence of other healthcare professions are not considered to a sufficient extent, although they have a profound impact on the healthcare situation. The latter in particular have a strong supportive function in regional healthcare.

**Objective:**

This study aimed to critically compare different parameters to evaluate the regional healthcare situation using the example of ophthalmologic care.

**Material and methods:**

For this purpose a score was calculated for the districts of the Hamburg metropolitan region based on the exemplary parameters “density of ophthalmologists with orthoptic focus on a district level (offer/100,000 inhabitants)”, “percentage of population with public transport accessibility to next ophthalmologist <30 min on a district level”, “percentage of population with public transport accessibility to next ophthalmologist with orthoptic focus <30 min on a district level” and compared with the results of the adjusted supply rate.

**Results:**

While the adjusted supply rate could not identify any case of undersupply in any of the districts, the other included parameters sometimes revealed clear differences in supply.

**Conclusion:**

The healthcare situation is heterogeneous by consideration of different parameters. This needs to be taken into account when assessing the regional healthcare situation for requirement planning.

## Hintergrund

Gesundheitsfachberufe wie die der Orthoptisten nehmen inzwischen eine tragende Entlastungsrolle niedergelassener Augenärzte ein, werden in der Bedarfsplanung jedoch nicht integriert betrachtet. Auch andere Parameter wie Erreichbarkeitsverhältnisse des öffentlichen Personennahverkehrs (ÖPNV) nehmen trotz Hinweisen des aktuellen Gutachtens zur Weiterentwicklung der Bedarfsplanung [16] in der Bewertung der ambulanten Versorgungssituation weiterhin eine eher untergeordnete Rolle ein [3].

Diese Untersuchung zielt darauf ab, dem korrigierten Versorgungsgrad bislang noch nicht in ausreichendem Maße in der Bedarfsplanung integrierte Parameter gegenüberzustellen und einen Ansatz aufzuzeigen, die Versorgungssituation neu zu bewerten.

## Methodik/Daten

Für die Untersuchung wurde sich auf die Metropolregion Hamburg fokussiert, da diese infrastrukturell heterogen ausgestattet ist und ein breites Versorgungsspektrum abbildet.

Datengrundlage aller Parameter sind aktuelle Standortdaten zu den niedergelassenen Augenärzten der Landesärztekammern Hamburg, Schleswig-Holstein, Niedersachsen (Stand: 2015) und Mecklenburg-Vorpommern (Stand: 2017) [7–10]. Es wurde in einem mehrschrittigen Verfahren geprüft, ob an den Praxisstandorten ein über das grundlegende Maß hinausgehendes orthoptisches/kinderophthalmologisches Angebot erkennbar war: Im ersten Schritt wurden die Praxisstandorte mit den Adressdatensätzen der Praxissuche („Augenarzt mit Orthoptist/‑in“) auf der Homepage des Berufsverband Orthoptik Deutschland e. V. (Stand 2015) abgeglichen. Im zweiten Schritt wurden die Praxishomepages nach den Schlagworten „Sehschule“, „Kindersprechstunde“ und „Orthoptist/-in“ durchsucht. Im dritten Schritt wurden die Namen der Praxisinhaber im Internet recherchiert und die gefundenen Einträge ebenfalls auf die Einträge „Sehschule“, „Kindersprechstunde“ und „Orthoptist/-in“ untersucht. Waren die untersuchten Hinweise zu finden, wurde der Praxisstandort als Standort mit orthoptischem Angebot klassifiziert.

Weitere relevante Qualitätsmerkmale wie Mindestanteil an Patienten im Kindesalter konnten im Rahmen dieser Untersuchung nicht berücksichtigt werden.

Als Berechnungsgrundlage der weiterführenden Analysen wurden die Geodaten des Bundesamtes für Kartographie und Geodäsie (administrative Grenzen; Stand: 2016), die LAEA-Rasterdaten (100 × 100 m) mit Bevölkerungszahlen (Zensus 2011) der Statistischen Ämter des Bundes und der Länder [15] sowie ein routingfähiges OpenStreetMap-Netz berücksichtigt (Stand: 2014) [11].

Folgende Parameter zur Abbildung der aktuellen augenärztlichen Versorgungssituation wurden errechnet, in einem Score „Versorgung“ zusammengeführt und mit dem korrigierten Versorgungsgrad verglichen:

- Dichte der Arztstandorte mit orthoptischem Angebot (Angebote/100.000 Einwohner) auf Kreisebene,
- Bevölkerungsanteil mit ÖPNV-Erreichbarkeit zum nächstgelegenen Augenarzt <30 min auf Kreisebene,
- Bevölkerungsanteil mit ÖPNV-Erreichbarkeit zum nächstgelegenen Augenarzt mit orthoptischem Angebot <30 min auf Kreisebene.

### Parameter

#### Korrigierter Versorgungsgrad

Die ambulante augenärztliche Versorgung wird über den sog. korrigierten Versorgungsgrad der aktuellen Bedarfsplanungs-Richtlinie des Gemeinsamen Bundesausschusses (GBA) geregelt [3]. Der korrigierte Versorgungsgrad wurde auf Basis der Grundrechenschritte aus der Anlage 4.2.2 der Bedarfsplanungs-Richtlinie für das Referenzjahr 2014 berechnet. Liegt im Ergebnis der korrigierte Versorgungsgrad bei über 110 %, so wird eine Überversorgung konstatiert. Zwischen 90 und unter 110 % liegt offiziell eine Regelversorgung vor, und unter 50 % wird von einer Unterversorgung ausgegangen [3]. Zur Beurteilung der augenärztlichen Versorgungssituation werden ausschließlich niedergelassene Augenärzte (Vertragsärzte) berücksichtigt. Datengrundlage sind neben den aktuellen Arztstandorten Bevölkerungsdaten von 2013.

#### Dichte

Die Dichte der Arztstandorte mit orthoptischem Angebot (Angebote/100.000 Einwohner) wird auf die Gesamtbevölkerung bezogen (Stand: 2013). Grundlage bilden die aktuellen Standortdaten der Ärztekammern Hamburg, Mecklenburg-Vorpommern, Schleswig-Holstein und Niedersachsen sowie die Bevölkerungszahlen auf Kreisebene [12].

#### Erreichbarkeitsauswertungen

Erreichbarkeitsauswertungen wurden mit der GIS-Software ArcMap 10.3.1 (©ESRI, Inc., Redlands, CA, USA) und der darin enthaltenen Funktionalität des Network Analyst vorgenommen. Diese erlaubt Erreichbarkeitsanalysen auf Basis eines routingfähigen Straßen- bzw. ÖPNV-Netzes. Als „Startpunkte“ wurden die bei den Ärztekammern recherchierten und geokodierten Arztstandorte verwendet. Ausgehend von diesen, wurden Erreichbarkeitsisochronen gebildet, die für jeden Arztstandort die Erreichbarkeit in Form der Wegezeiten darstellen. Daraus ergeben sich die sog. Service Areas. In einem zweiten Schritt wurden diese Flächen mit den Mittelpunkten der 100 × 100 m LAEA-Rasterzellen [15] verschnitten, denen auf Basis des Zensus 2011 Bevölkerungsanteile zugeordnet werden konnten. Auf diese Weise wurde ermittelt, wie hoch der Anteil der Bevölkerung ist, der den nächstgelegenen Augenarzt (mit/ohne orthoptisches Angebot) innerhalb von 30 min Wegezeit erreicht. Bei dem Schwellenwert von 30 min wurde sich an Mindeststandards zu anderen medizinischen Infrastrukturen (Kliniken oder Hausärzte) orientiert [17], da für die fachärztliche Versorgung derartige Mindeststandards einer wohnortnahen Versorgung noch nicht vorliegen [2]. Der Fokus wurde hierbei auf die ÖPNV-Erreichbarkeit gelegt, da zum einen nicht jeder den Zugang zu einem Pkw hat und zum anderen Studien darauf hinweisen, dass Fachärzte mit dem Pkw meist deutlich unter 30 min erreichbar sind, sodass die Pkw-Erreichbarkeit in diesem Kontext nicht weiter betrachtet wurde [2].

### Vergleich der Parameter: Score „Versorgung“ und korrigierter Versorgungsgrad

Zur Vergleichbarkeit der Parameter mit dem korrigierten Versorgungsgrad wurde ein Score „Versorgung“ (ohne Versorgungsgrad) gebildet. Dazu wurden die drei Dimensionen Arztstandorte mit orthoptischem Angebot je 100.000 Einwohner, der Bevölkerungsanteil mit einer ÖPNV-Erreichbarkeit zum nächstgelegenen Orthoptisten <30 min sowie zum nächstgelegenen Augenarzt <30 min zunächst auf 100 % standardisiert (Anteil am jeweiligen Mittelwert). Anschließend wurden in Anlehnung an den korrigierten Versorgungsgrad die Klassengrenzen jeweils bei ≤90 %, >90 % bis 110 %, >110 % gesetzt und entsprechend Punkte vergeben (0 = unterdurchschnittlich, 1 = durchschnittlich, 2 = überdurchschnittlich). Zur Ermittlung des Scores „Versorgung“ wurde die mittlere Punktzahl errechnet, sodass minimal (wenigstens) 0,0 und maximal (höchstens) 2,0 Punkte erreicht werden.

### Grenzziehung Metropolregion

Die Metropolregion wurde 2017 um einzelne Teilräume erweitert. Hierzu gehören der Altkreis Parchim des neuen Landkreises Ludwigslust-Parchim und die Landeshauptstadt Schwerin. Im Rahmen dieser Untersuchung wurde jedoch die Grenzziehung vor der genannten Erweiterung berücksichtigt, da das routingfähige Straßennetz auf den alten Gebietsstand hin zugeschnitten wurde.

Alle kartografischen Darstellungen der Ergebnisse wurden auf Basis der Leitlinien der Guten Kartographischen Praxis (GKPiG) erzeugt [1].

## Ergebnisse

### Parameter

In der Metropolregion Hamburg wird die augenärztliche Grundversorgung derzeit mit 338 niedergelassenen Augenärzten in 251 Praxisstandorten sichergestellt. Ein Großteil der Standorte (47 %) liegt im Hamburger Stadtgebiet; 189 dieser Arztstandorte verfügen über ein explizites orthoptisches Angebot [7–10].

#### Korrigierter Versorgungsgrad

**Figure 1:**
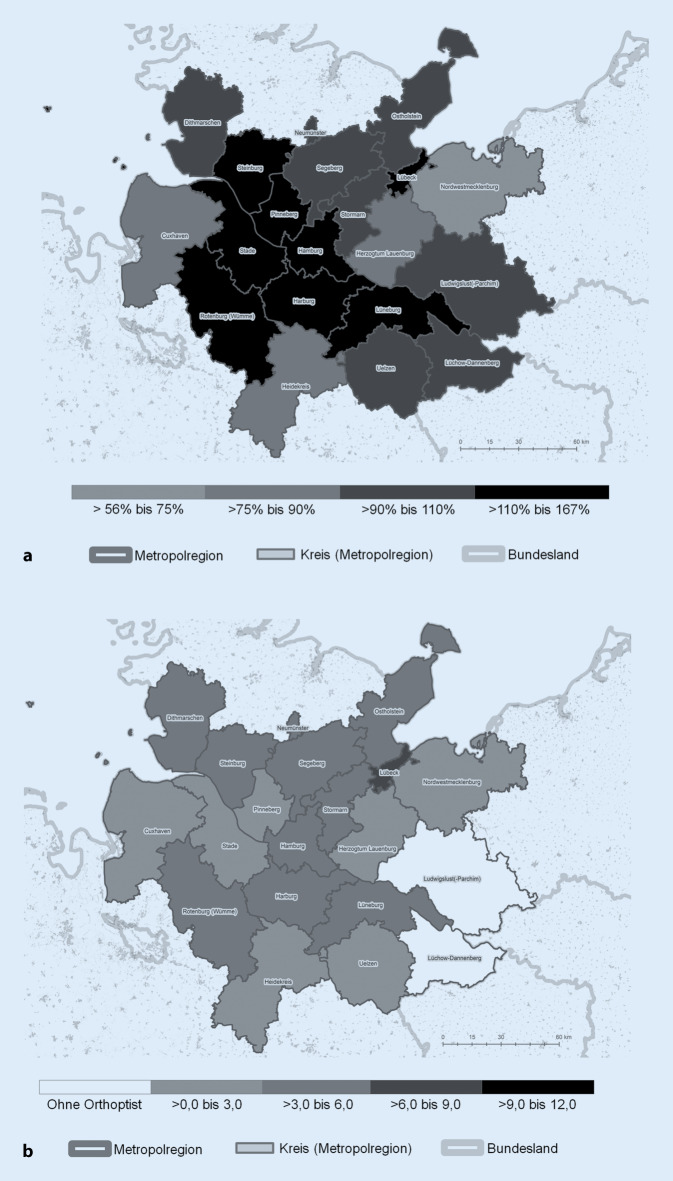

Der korrigierte Versorgungsgrad zeigt für die Metropolregion ein heterogenes Bild (Abb. 1a). Während insbesondere die urbanen Zentren Lübeck und Hamburg hinsichtlich der augenärztlichen Versorgung auf Basis der Bedarfsplanung deutlich überversorgt sind, sind andere Kreise mit >75 % bis 90 % bzw. Nordwestmecklenburg mit 56 % noch offiziell regelversorgt. Offiziell gilt keiner der Teilräume laut GBA als unterversorgt.

#### Dichte

Wird die Dichte der Arztstandorte mit orthoptischem Angebot betrachtet (Abb. 1b), so zeigt sich in beiden Fällen ein West-Ost-Gefälle. Während die westlichen Teilräume der Metropolregion wie Cuxhaven, Steinburg und Rotenburg eine vergleichsweise hohe Angebotsdichte aufweisen, zeigen alle östlichen Teilräume ein eher durchschnittliches Angebot-Einwohner-Verhältnis (>3,0 bis 6,0). Mit Ludwigslust und Lüchow-Dannenberg gibt es demgegenüber sogar Teilräume, denen die orthoptische Versorgung gänzlich fehlt. Im Vergleich dazu fällt der Standort Lübeck mit einer vergleichsweise hohen orthoptischen Angebotsdichte auf.

#### Erreichbarkeitsauswertungen

**Figure 2:**
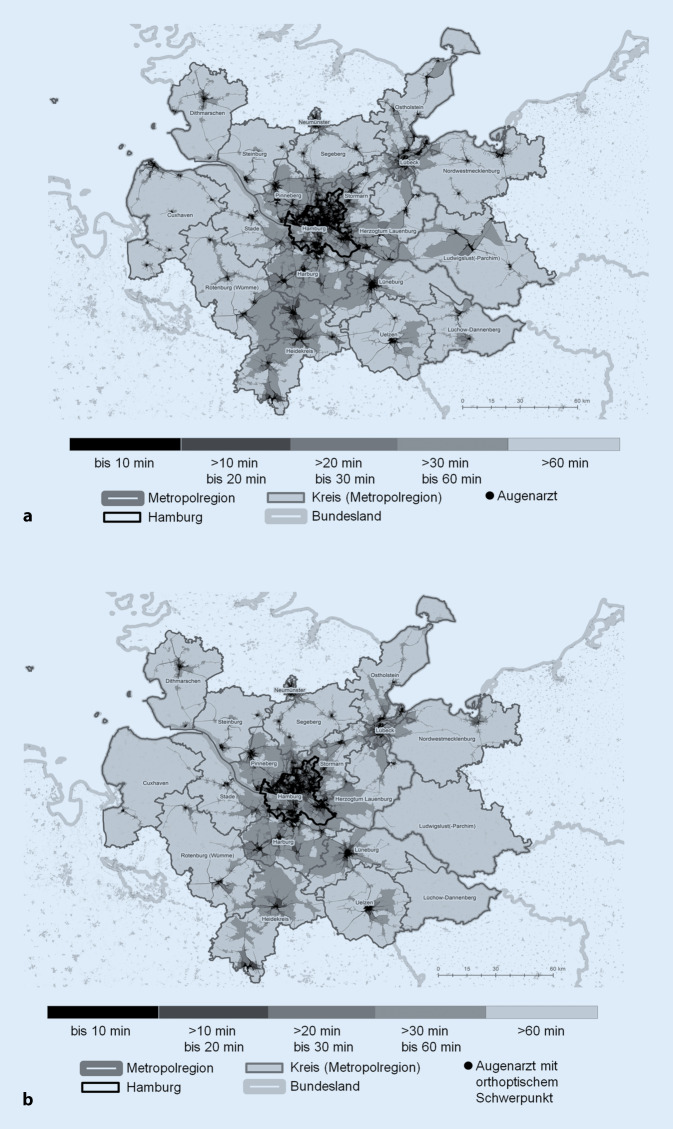

Auf Basis der aktuellen Praxisstandorte und der Praxisstandorte mit orthoptischem Angebot wurden die Erreichbarkeitsanalysen für den ÖPNV durchgeführt. Für weite Teile der Bevölkerung in der Metropolregion sind Fahrzeiten von über 60 min zum nächsten Augenarzt (z. B. Steinburg: 51 %, Dithmarschen: 57 %) einzuplanen (Abb. 2a). Bei den Praxisstandorten mit orthoptischem Angebot fällt die Versorgungssituation aufgrund der Arztstandortkopplung ähnlich, wenngleich für einzelne Teilräume z. T. deutlich schlechter aus (Abb. 2b). Dies trifft insbesondere auf die eher ländlich geprägten und dispers besiedelten Teilräume zu, die mit einer im Vergleich zu den urbanen Kommunen der Metropolregion schlechteren ÖPNV-Verbindungsqualität konfrontiert sind. In etwa der Hälfte der Kreise können weniger als 50 % der Bevölkerung einen Augenarzt innerhalb von 30 min erreichen.

### Vergleich der Parameter: Score „Versorgung“ und korrigierter Versorgungsgrad

**Figure 3:**
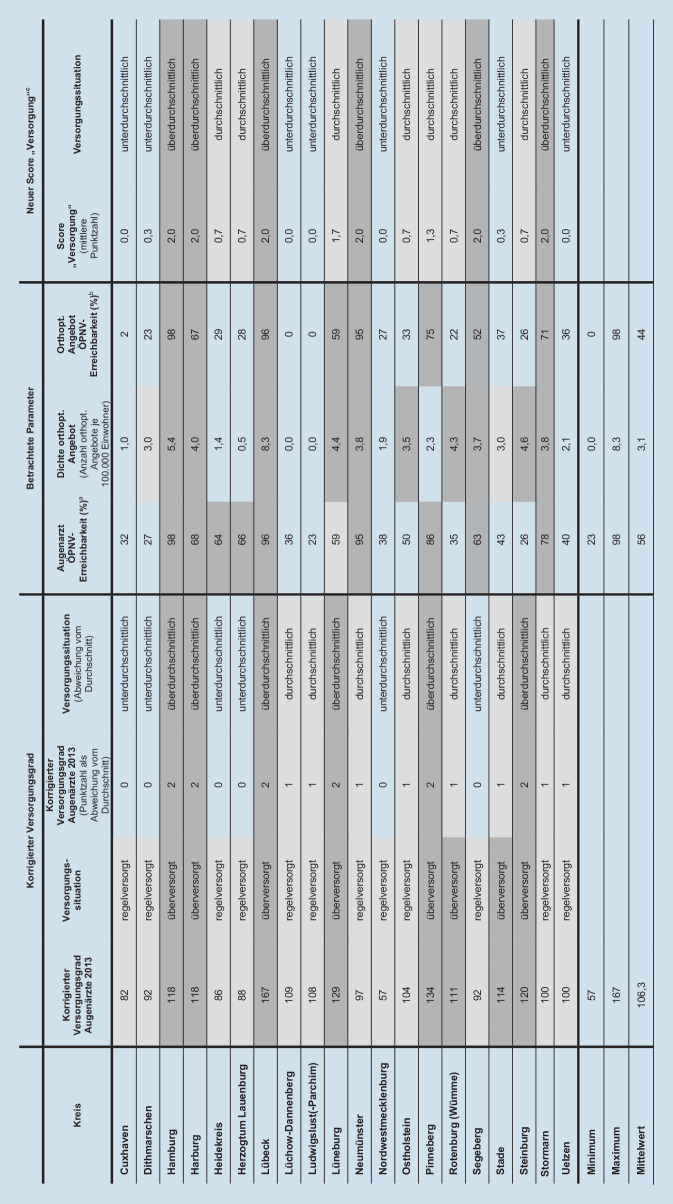

Ein Vergleich aller einbezogenen Parameter zur Abbildung der aktuellen Versorgungssituation offenbart, dass ein korrigierter Versorgungsgrad nicht gezwungenermaßen mit der Dichte an orthoptischen Angeboten oder Erreichbarkeitsverhältnissen zusammenhängt (Abb. 3). Insbesondere in laut korrigiertem Versorgungsgrad regelversorgten Kreisen wie Dithmarschen, Lüchow-Dannenberg, Ludwigslust(‑Parchim), Nordwestmecklenburg und Uelzen fallen die genannten Parameter unterdurchschnittlich aus. Auffallend sind darüber hinaus die Ergebnisse einzelner Kreise mit einem korrigierten Versorgungsgrad <90 %, die als offiziell regelversorgt gelten. Die urbanen Zentren Hamburg und Lübeck zeichnen sich durch durchweg überdurchschnittliche Parameterausprägungen aus.

## Diskussion

Bereits für andere Facharztgruppen belegen Studien eine Diskrepanz zwischen Erreichbarkeitsverhältnissen und korrigiertem Versorgungsgrad [2]. Eine nach der Bedarfsplanung offiziell bedarfsgerechte medizinische Versorgung ist demnach nicht gleichbedeutend mit (über)durchschnittlichen Erreichbarkeitsverhältnissen. Auch andere Studien offenbaren Limitationen des korrigierten Versorgungsgrades, die Versorgungssituation realitätsnah abzubilden [4, 6, 18]. Insbesondere das aktuelle Gutachten zur Weiterentwicklung der Bedarfsplanung [16] lieferte jüngst ein umfassendes Bild zu versorgungsrelevanten Faktoren im Gesundheitsbereich, auf dessen Basis bereits anteilig, aber nicht allumfassend Änderungen in der Bedarfsplanungs-Richtlinie umgesetzt wurden. Die vorliegende Untersuchung stützt die Ergebnisse dieser Studien und zeigt auch hier, dass eine offiziell bedarfsgerechte ambulante augenärztliche Versorgung nicht zwangsläufig mit einem (über)durchschnittlichen Zugang zu Praxisstandorten (mit/ohne orthoptisches Angebot) einhergeht. Insbesondere in den östlichen Teilräumen der Metropolregion wird diese Diskrepanz sichtbar. Auffallend ist dabei, dass Praxisstandorte mit orthoptischem Angebot insbesondere dort fehlen, wo der Bedarf aufgrund der Bevölkerungsentwicklung und altersstruktureller Verschiebungen tendenziell zunehmen könnte. Diese demografiebedingten Bedarfsveränderungen erfordern eine Anpassung der Ressourcen [13, 14] und sprechen den Gesundheitsfachberufen vor dem Hintergrund begrenzter Niederlassungsmöglichkeiten durch die Bedarfsplanung in Zukunft eine wachsende Bedeutung zu [3, 5, 14, 19].

Die aktuelle Bedarfsplanung hat darauf trotz Hinweisen aus dem aktuellen Gutachten zur Bedarfsplanung noch nicht im ausreichenden Maße reagiert. So zeigt bereits die Datenlage zu versorgungsrelevanten Gesundheitsfachberufen erhebliche Lücken und lässt keine verlässlichen Aussagen zur Versorgungssituation diesbezüglich zu. Eine zentral geregelte und zusammen mit der ärztlichen Versorgung integrierte Erhebung sollte anvisiert werden.

Auch bei der Beurteilung der Versorgungssituation zeigt diese Untersuchung erneut und reiht sich damit in die Ergebnisse anderer Untersuchungen ein, dass das Spektrum zur Abbildung der Versorgungssituation trotz der jüngsten Anpassungen noch nicht ausgeschöpft ist und seitens der Bedarfsplanung stetig erweitert werden sollte. In dem Zusammenhang muss darauf hingewiesen werden, dass der im Rahmen dieser Untersuchung errechnete Score nur eine Auswahl von möglichen Versorgungsparametern berücksichtigt und damit keinen alternativen Versorgungsgrad darstellt. Relevante Aspekte wie das Alter der Ärzte oder Wartezeiten sowie ergänzende klinische Versorgungsmodelle konnten beispielsweise nicht mit einbezogen werden.

Zudem bezieht sich der Vergleich der einzelnen Regionen nur auf die Werte innerhalb der Metropolregion (Abweichung vom Durchschnitt innerhalb der Metropolregion) und kann nicht exemplarisch für die Bundesrepublik Deutschland stehen. Die Frage bleibt also offen, wann von einer nicht ausreichenden Anzahl an Praxisstandorten mit orthoptischem Angebot, schlechten oder unzumutbaren Erreichbarkeitsverhältnissen sowie generell von einer unzureichenden Versorgungssituation gesprochen werden kann. Was für einzelne Bevölkerungsgruppen oder Regionen beispielsweise noch eine annehmbare Entfernung darstellt, kann für andere schon eine unüberwindbare Distanz sein. Da spezielle orthoptische Angebote eher von Kindern bzw. jungen Familien in Anspruch genommen werden, ist die Verfügbarkeit eines Pkw als höher einzuschätzen und die Problematik einer schlechteren ÖPNV-Anbindung eher geringer ausgeprägt. Demgegenüber führen typische altersabhängige Augenerkrankungen eher zum Verlust der Fahrtüchtigkeit, und eine schlechte ÖPNV-Anbindung ist dann problematischer. Jedoch sind gerade auch im höheren Lebensalter Erkrankungen möglich, die Diplopie verursachen und dadurch zur Fahruntauglichkeit führen. Gerade diese Krankheitsbilder werden wiederum dann in Praxen mit orthoptischem Angebot versorgt. Die Frage nach bevölkerungsgruppenspezifischen Mindeststandards für die fachärztliche Versorgung muss in diesem Kontext gestellt werden.

## Fazit für die Praxis

- Für einen Großteil der Bevölkerung in den eher ländlich geprägten Teilräumen der Metropolregion Hamburg sind Angebote der augenärztlichen Versorgung mit öffentlichen Verkehrsmitteln nur schwer zu erreichen. Andere Zugangsmöglichkeiten insbesondere für immobile Patienten sollten geprüft werden.
- Die regionale Versorgung kann auf Basis verschiedener Parameter unterschiedlich bewertet werden. Bei der Beurteilung der Versorgungssituation sollten an der Grundversorgung teilnehmende Gesundheitsfachberufe sowie ÖPNV-Erreichbarkeiten stärker mit berücksichtigt werden (Mindeststandards).
- Es ist davon auszugehen, dass der augenärztliche Versorgungsbedarf in Zukunft weiter zunehmen wird. Gesundheitsfachberufe können dabei ein wichtiges Entlastungspotenzial in der medizinischen Grundversorgung darstellen. Entsprechende Datengrundlagen sollten geschaffen werden.
